# Comment on Castien et al. (2018) pressure pain thresholds over the cranio-cervical region in headache - a systematic review and meta-analysis

**DOI:** 10.1186/s10194-018-0859-x

**Published:** 2018-04-13

**Authors:** Kerstin Luedtke, Tibor Szikszay, Wacław Adamczyk, Arne May

**Affiliations:** University of Luebeck, Medical section, Academic Physiotherapy, Luebeck, Germany

The recently published systematic review by Castien et al. [1] is summarising data in a field of highly ambiguous reporting. Pain thresholds in headache patients have been investigated by a large number of researchers with inconsistent results and it is certainly important to summarise and evaluate all data in a systematic review and meta-analysis. However, the present review can only partially shed light on the issue, due to a number of rather grave methodological shortcomings.

The underlying concept is, that especially the two primary headache types, TTH and migraine, show a component of (peripheral or central) sensitization. There are two major difficulties for researchers trying to clarify the presence or absence of sensitization:

- The gold standard is the quantitative sensory testing (QST) protocol [2]. The test protocol is time consuming and, if conducted completely, the mere number of parameters, which have to be corrected for multiple testing, requires sample sizes that cannot feasibly be recruited at a single headache clinic. The reported QST results for any type of pain (not exclusively headache) usually describes that only a small number of tests (e.g. heat pain thresholds and pressure pain thresholds) are significantly different between patients and controls, while the majority of tests do not show any significant differences. Such results are difficult to interpret. Only using or reporting a few tests, e.g. like in this case pressure pain thresholds, does not give the full picture of the disorder and cannot distinguish between sensitization and no sensitization. Pin-Prick hyperalgesia or heat hyperalgesia would have given a clearer picture.
- The general opinion in migraine is, that sensitization mainly occurs as a part of the attack and is absent interictally [3]. It is therefore mandatory to distinguish between data measured during or outside of attacks. Pooling data from both time-points ignores basic pathophysiological knowledge and additionally is likely to result in an underestimation of the effect size.

Looking at all QST parameters, while informative and more likely to clarify sensitization, would require a large systematic review. It is understandable to focus on only one aspect of QST, however, this should be discussed as a major limitation.

Distinguishing between ictal and interictal measurements is certainly a difficult task for a systematic review team, since this information is rarely provided in studies. Again, this should be added as a limitation and maybe also included in the discussion to stimulate more precise reporting in future studies.

Two of the main points can therefore not be answered by the present systematic review due to feasibility and reporting problems. There are some further methodological issues we would like to highlight, since these might alter,in this case even completely change the results of the meta-analysis:

- The literature search has a cut-off date reported as August 2015. This is more than 2.5 years ago and an updated search would have identified at least one additional study [4]
- The search was limited to 3 databases (the protocol in PROSPERO says that originally 4 databases were anticipated for the search) and did not include (baseline data from) randomised controlled trials. Maybe this was the reason why so many studies were not included in the review that report pressure pain thresholds in headache populations [5–7]?

Even without additional papers, the data extraction Table (7 pages) is difficult to read. The results could have been reported in two columns, e.g. results from patients and results from controls (or painful versus non-painful side), to organize the amount of information in this one column. Some data is presented in the incorrect column (e.g. Fernandez-des-las-penas (number 27): F/M is presented in the column for blinding). Blinding is already part of the risk of bias table (why was none of the tools used that are recommended by the Cochrane Collaboration?) and could have been excluded from the data extraction table to save some space.

Last but not least, what happened to the five papers reported in the abstract that are not included in the main body of the paper? The abstract reports 22 selected studies, while Fig. 1 and the main text report only 17 included studies. It appears that the abstract may not have been updated after the exclusion of these 5 studies.

Was the protocol changed retrospectively? And if so: Why? The protocol registered in PROSPERO does not answer this question, since the review was registered retrospectively which is in itself highly problematic. Why is the PROSPERO registration number not stated in the report?

We were looking forward to read this paper and to finally find some clarification on the issue of the presence or absence of pressure pain thresholds maybe indicating sensitization. Unfortunately, this review does not rise to this challenge. Hopefully there will soon be an updated systematic review, ideally including all QST items, and distinguishing between ictal and interictal measurements for the migraine population and with more rigour regarding methods and reporting.
